# The Army and Territorial Nurses' Cape

**Published:** 1915-11-13

**Authors:** A. W. Gill

**Affiliations:** Lady Superintendent of Nurses. The Royal Infirmary, Edinburgh


*To the Editor of* THE HOSPITAL.

### The Army and Territorial Nurses' Cape.

*To the Editor of* THE HOSPITAL.

SIR,—As the current number of THE HOSPITAL has reached us three days late your attention may already have been drawn to the fact that the nurses' cape is mentioned in Sir Edward Cook's biography of Miss Nightingale (vol. i., pp. 186 and 187). The cape was part of the uniform as shown by the contemporary description of a Roman Catholic sister, quoted by the author: "The Nightingale nurses in the East wore 'grey tweed wrappers, worsted jackets, with caps and short woollen cloaks.'" Sir Edward Cook then quotes from the journal of the Royal Army Medical Corps: "The red uniform cape worn by the ladies of the Queen Alexandra's Imperial Military Nursing Service is modelled on that originally introduced by Florence Nightingale," etc.—I am, yours faithfully, A. W. GILL,

Lady Superintendent of Nurses.

The Royal Infirmary, Edinburgh, November 4, 1915.

[We are grateful to Miss Gill for her letter. The statement we published as to Sir Edward Cook's biography came to us from an authoritative source, which we were entitled to accept without question.—ED. THE HOSPITAL.]

